# Angioedema in the emergency department: clinical presentation and outcomes

**DOI:** 10.1186/1710-1492-10-S1-A34

**Published:** 2014-03-03

**Authors:** Sarah L Felder, R M Curtis, Ian Ball, Rozita Borici-Mazi

**Affiliations:** 1 School of Medicine, Queen’s University, Kingston, Ontario, K7L 3N6, Canada; 2 Department of Emergency Medicine, Queen’s University, Kingston, Ontario, K7L 3N6, Canada; 3 Division of Allergy/Immunology, Department of Internal Medicine, Queen’s University, Kingston, Ontario, K7L 3N6, Canada

## Rationale

Angioedema is an acute, potentially life-threatening presentation with multiple mechanistically distinct causes. We hypothesized that the clinical features of angioedema correlate with the cause of angioedema and may predict the outcomes of angioedema in the emergency department (ED).

## Methods

A retrospective data review of all ED visits to two academic tertiary care centers over the period of July 1, 2007 to March 31, 2012 was conducted. Records selected for full review met the inclusion criteria of documented visible swelling and one of the ICD-10 diagnostic codes for anaphylactic shock, angioneurotic edema, allergy unspecified, defects in the complement system, or unspecified drug adverse effect. Age, sex, cause and site of swelling, comorbidities, medications, allergies, admissions, etc. were collected via a standardized form. Bivariate and multivariate analyses were performed using chi-square tests. Potential predictors for admission were identified using a multivariate logistic regression model. A p value less than 0.05 was considered significant. This study was approved by Queens University Ethics Committee.

## Results

Medical records from 527 ED visits by 455 patients were included in the study, and 21 patients were admitted. Angioedema was encountered at annual rate of 10 per 10000 ED visits. Patients who presented with urticaria (29.8%) were significantly more likely to present with lip swelling (*p*=0.001) and extremity swelling (*p*=0.008), while the absence of urticaria correlated with tongue swelling (*p*=0.001). The mean duration of stay in ED was significantly longer in patients with urticaria (*p*<0.001), but the presence of urticaria did not predict admission. A probable cause was identified in 48.8% of visits. Periorbital angioedema was associated with environmental, contact, and insect sting allergy (*p*<0.001). 58.3% of patients with angioedema due to drug allergy had lip angioedema (*p*=0.032). C1 esterase inhibitor deficiency was most frequently associated with a history of previous episodes. Several factors were found to predict admission, including NSAID-induced angioedema (OR=15.3), epinephrine treatment (OR=8.34), hypotension (OR=15.7), multiple site angioedema (OR=4.25), pharyngeal angioedema (OR=1.23), and tongue angioedema (OR=4.62).

## Conclusions

This large cohort retrospective review confirms cause-clinical associations in angioedema and demonstrates novel predictors of morbidity, with implications in clinical practice.

